# Reactivating aberrantly hypermethylated *p15 *gene in leukemic T cells by a phenylhexyl isothiocyanate mediated inter-active mechanism on DNA and chromatin

**DOI:** 10.1186/1756-8722-3-48

**Published:** 2010-11-29

**Authors:** Shaohong Jiang, Xudong Ma, Yiqun Huang, Yunlu Xu, Ruiji Zheng, Jen-Wei Chiao

**Affiliations:** 1 Zhangzhou Affiliated Hospital of Fujian Medical University, Zhangzhou, Fujian Province, China; 2 School of Pharmacy, Fujian Medical University, Fuzhou, Fujian Province, China; 3 Department of Medicine, New York Medical College, Valhalla, NY 10595, USA

## Abstract

**Background:**

We have previously demonstrated that phenylhexyl isothiocyanate (PHI), a synthetic isothiocyanate, inhibits histone deacetylases and remodels chromatins to induce growth arrest in HL-60 myeloid leukemia cells in a concentration-dependent manner.

**Methods:**

To investigate the effect of PHI, a novel histone deacetylases inhibitor (HDACi), on demethylation and activation of transcription of *p15 *in acute lymphoid leukemia cell line Molt-4, and to further decipher the potential mechanism of demethylation, DNA sequencing and modified methylation specific PCR (MSP) were used to screen *p15*-M and *p15*-U mRNA after Molt-4 cells were treated with PHI, 5-Aza and TSA. DNA methyltransferase 1 (DNMT1), 3A (DNMT3A), 3B (DNMT3B) and *p15 *mRNA were measured by RT-PCR. P15 protein, acetylated histone H3 and histone H4 were detected by Western Blot.

**Results:**

The gene *p15 *in Molt-4 cells was hypermethylated and inactive. Hypermethylation of gene *p15 *was attenuated and *p15 *gene was activated de novo after 5 days exposure to PHI in a concentration-dependent manner. DNMT1 and DNMT3B were inhibited by PHI (P < 0.05). Alteration of DNMT3A was not significant at those concentrations. Acetylated histone H3 and histone H4 were accumulated markedly after exposure to PHI.

**Conclusion:**

PHI could induce both DNA demethylation and acetylated H3 and H4 accumulation in Molt-4 cells. Hypermethylation of gene *p15 *was reversed and *p15 *transcription could be reactivated de novo by PHI.

## Background

The major epigenetic transcriptional controls involved in gene silencing are DNA methylation and covalent modification of histone proteins. Transcriptional silencing of genes, due to hypermethylation of CpG islands in the promoter region of genes, has been reported in nearly every type of human tumors. A broad spectrum of genes are frequently hypermethylated in cancers, including those associated with cell cycle regulation, detoxification, tumor suppression, and apoptosis etc. DNA methylation is catalyzed by DNA methyltransferases (DNMTs), of which three active enzymes have been identified in mammals, namely DNMT1, DNMT3A and DNMT3B. In developmental processes of the mouse, DNMT1 is responsible for maintaining pre-existing methylation patterns during DNA replication, while DNMT3A and DNMT3B are required for initiation of de novo methylation. DNMT3A expression is ubiquitous, but DNMT3B is present in the cells at low levels except in testes, thyroid, and bone marrow[1]. DNMTs play roles in gene silencing by acting as transcriptional repressors themselves, or by serving as binding scaffolds for transcriptional repressors, histone deacetylases and histone methyltransferases. Thereby, DNMTs can establish gene silencing independent of their catalytic activities[2-4]. In human leukemia cells, the DNMTs are found to be aberrantly over-expressed[5,6]. Histone proteins assemble into nucleosomes, which function as both DNA packaging units and transcriptional regulators. The amino-terminal tails of histones protrude from the nucleosome and they are subjected to covalent modifications such as acetylation, methylation and phosphorylation. The different types of histone modifications have been linked with distinct functions. Modifications to histones influence chromatin structure, and ultimately gene transcription, including those coding for tumor suppressor proteins. One of the key histone modification that control gene transcription is acetylation, which is regulated by two opposing enzymatic activities (histone acetyltransferases [HATs] and histone deacetylases [HDACs])[7]. HATs are in charge of histone acetylation, leading to the relaxation of chromatin structure and transcriptional activation of genes, while HDACs are in charge of histone deacetylation, which is associated generally with chromatin condensation and transcriptional repression[8].

The gene *p15 *(*INK4b *or *MTS2*) is a candidate tumor suppressor with structural and functional similarity to the *p16 *gene. This gene recognizes cyclin-dependent kinases CDK4 and CDK6, and induces G1 arrest of the cell cycle by competing with cyclin D for binding with CDK4. Specific deletions of *p15 *sequences have been found in only a few cases of leukemia and lymphomas. In contrast, the *p15 *gene is preferentially hypermethylated at a 5'-CpG island, which has been shown to be associated with loss of transcription of this gene in leukemia cells [9,10]. Furthermore, aberrant *p15 *methylation seems to have important prognostic implications for risk assessment because patients with *p15 *methylation have overall shortened survival [11,12].

We have previously demonstrated that phenylhexyl isothiocyanate (PHI), a man-made isothiocyanate, inhibits histone deacetylases and remodels chromatins to induce growth arrest in HL-60 myeloid leukemia cells in a concentration-dependent manner[13]. Recent research has described that this class of small chemicals, either present naturally in cruciferous vegetables or man-made are potential chemopreventive agents. In animal models, PHI was effective against tumorigenesis of esophagus, leukemia, and carcinomas of lung and prostate[14-17]. The major mechanism includes the induction of growth arrest and apoptosis in tumor cells[18,19]. Since we have demonstrated that PHI is an inhibitor of HDACs, its effects on *p15 *activity in leukemia cells has been a subject of investigation. In this paper we demonstrated that PHI has a dual effect on DNA methylation and histone acetylation in leukemic T cells Molt-4. The cross-talk on the DNA and chromatin resulted in demethylating the CpG island of *p15*, which is inactivated due to hypermethylation, and recovered the unmethylated *p15 *for transcriptional activation. An inter-active mechanism, involving a down-regulation of the enzymes DNMTs and up-regulating the histone acetyltransferase P300/CBP and histone acetylation, is revealed.

## Methods

### Cell cultures

PHI, greater than 98% pure, was purchased from LKT Lab (St. Paul, MN). Human acute lymphatic leukemia cell line Molt-4 was obtained from China Center for Type Culture Collection (CCTCC). Cells were maintained in RPMI-1640 medium supplemented with 10% heat-inactivated fetal calf serum and maintained at 37°C in humidified atmosphere containing 5% CO_2_. Cells in exponential growth were exposed to PHI prepared in 75% methanol[13] at various concentrations for 5 days. The control cultures were supplemented with the methanol-containing medium. Some cell cultures were supplemented with 2 μM of 5-azacytidine (5-Aza), a known inhibitor of DNA methylation, or 1 μM of Trichostatin A (TSA), a known inhibitor of histone deacetylases, at various concentrations.

### Methylation specific PCR (MSP)

The genomic DNA from cultured cells was extracted and modified by bisulfate treatment for MS-PCR analyses[20]. DNA from cell cultures under different conditions was isolated with the Tissue/Cell Genomic DNA Isolation Kit (Pearl, China), employing the Wizard DNA Clean-Up System (Promega, USA), and amplified by PCR with two sets of gene promoter specific primer pairs that recognize the methylated (M) and the unmethylated (U) CpG sites. The primers for the methylated form of *p15 *(148pb) were gcgttcgtattttgcggtt (positive sense), and cgtacaataaccgaacgaccga (antisense). The primers for unmethylated form (154 bp) were tgtgatgtgtttgtattttgtggtt (positive sense) and ccatacaataaccaaacaaccaa (antisense). The amplification was performed in an Mastercycler unit **(**Eppendorf) under the program conditions as follows: 95°C for 5 min; then 40 cycles of 95°C for 45 sec, 60°C for 45 sec, 72°C for 45 sec; and finally 10 min at 72°C. The PCR products were visualized in GeneGenius (Syngene, British) by ethidium bromide staining in 2% agarose gels.

### Western blot analysis

The protein levels were determined by Western blot analysis as described previously[21]. Briefly, total proteins were prepared from each culture condition with a lysis buffer containing protease inhibitors, and the lysates collected after centrifugation at 4°C. The protein content of the lysates was determined with the Bradford protein assay. Protein lysate was subjected to SDS-PAGE, electrotransferred to nitrocellulose membrane, and immunoblotted with specific antibodies. The following antibodies were used for immunoblotting: anti-acetyl-histone H3 (lysines 9 and 14) (1:300 dilution, Upstate), anti-acetyl-Histone H4 (lysines 5, 8, 12, and 16) (1:300 dilution, Upstate), and anti-300/CBP (1:300 dilution, Upstate). Antibodies against P15, and a goat anti-rabbit with HRP conjugate secondary antibodies were purchased from Santa Cruz (USA). The reaction was visualized through the ECL system, and the protein levels were quantified with the anti-β-actin protein estimation assay.

### Reverse Transcription-Polymerase Chain Reaction (RT-PCR)

Total RNA was isolated using Trizol reagent (Invitrogen, USA) according to the manufacturer's instructions. One-microgram total RNA isolated was used for the first-strand cDNA synthesis with Reverse Transcription System (Promega, USA). cDNA was amplified using specific primer for *p15*, DNMT1, DNMT3A, DNMT3B in separate reactions (Table 1). β-actin was used as a loading control to ensure that cDNA was complete and Taq was deactivated in each reaction. The PCR products were visualized in GeneGenius (Syngene, British) by ethidium bromide staining in 1.6% agarose gels.

**Table 1:** PCR premiers for P15, DNMT1, DNMT3A, and DNMT3B mRNA

	sequence (5'-3')	extent (bp)	**anneal temperature (**°C)
actin1	F:gtggggcgccccaggcacca	517	Variable
	R:ctccttaatgtcacgcacgatttc		
actin2	F:ctacaatgagctgcgtgtggc	271	Variable
	R:caggtccagacgcaggatggc		
*p15*	F:tgggggcggcagcgatgag	451	56
	R:aggtgggtgggggtgggaaat		
DNMT1	F:accatcacatctcattttgc	238	56
	R:ggtttgacttcggagtctct		
DNMT3A	F:cacacagaagcatatccaggagtg	551	55
	R:agtggactgggaaaccaaataccc		
DNMT3B	F:aatgtgaatccagccaggaaaggc	190	55
	R:actggattacactccaggaaccgt		

F: Forward primer R: Reverse primer

cDNA was amplified using specific primer for, DNMT1

### Statistical analysis

The data was analyzed by statistical software SPSS13.0. Data measurement was presented as mean ± SD, from multiple independent experiments by homogeneity test for variance and test of normality. Results were evaluated by One-way ANOVA between groups. *P *< 0.05 was considered to be statistically significant.

## Results

### Demethylating *p15 *by PHI

**Figure 1:**
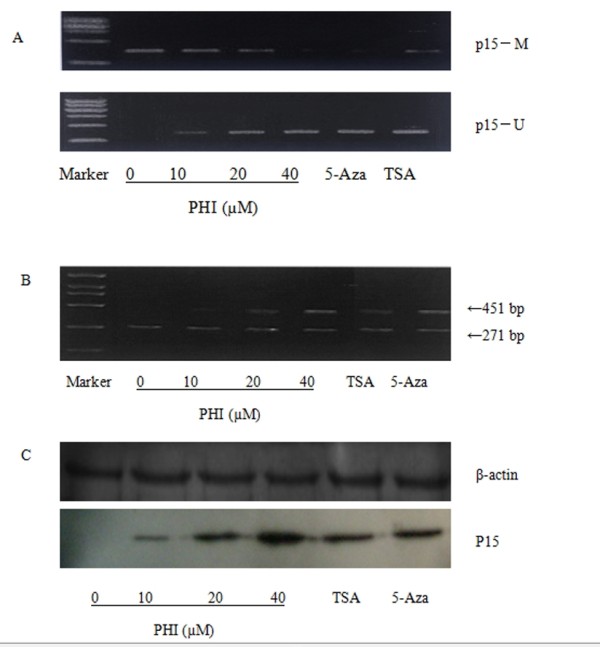
**PHI reversed hypermethylation of *p15 *and induced transcription activation**. **A**: PHI induced p15 hypomethylation with the decreasing of the methylated *p15 *(*p15*-M) and increase of the unmethylated form of *p15 *(*p15*-U). MS-PCR was performed using primers specific for *p15*-M and *p15*-U DNA forms of *p15 *as described in the methods. 5-Aza (2 μM) and TSA (1 μM) were used as controls for DNA hypomethylation. **B**: P15 mRNA levels were enhanced by PHI treatment. The expression levels of p15 mRNA were measured by RT-PCR. **C**: Up-regulation of *p15 *protein expression by PHI. P15 protein levels were detected by Western blotting, and β-actin was used as a loading control.

The DNA methylation status of *p15 *in human lymphatic leukemia T cells Molt-4 was evaluated by MS-PCR. Figure 1A showed the presence of methylated *p15 *(*p15*-M), and the unmethylated *p15 *(*p15*-U) was undetectable. After exposure of Molt-4 cells to PHI for 5 days, the methylated *p15 *was decreased, with the magnitude in positive relation to the PHI concentrations. The unmethylated *p15 *became detectable, replacing the decrease of methylated *p15*. The activity of 40 μM PHI to reverse *p15 *methylation was similar to that of 2 μM of 5-Aza, a known inhibitor of DNA methylation or 1 μM of TSA, which was shown in endometrial cancer cells as a demethylating agent as it reduced DNMT3B level and de novo DNMT activity[22].

The mRNA level of *p15 *in Molt-4 cells was examined without or with the exposure to PHI. Figure 1B showed the comparison of the levels of mRNA of *p15 *and β-actin. In the presence of PHI, the *p15 *mRNA expression was enhanced in a concentration-dependent manner. The ratios were: control (0.17 ± 0.12), PHI 10 μM (0.29 ± 0.14), PHI 20 μM (0.55 ± 0.07), PHI 40 μM (0.93 ± 0.13), TSA (0.65 ± 0.11), and 5-Aza (0.89 ± 0.13). The expression levels of p15 mRNA mediated by PHI were statistically significantly (*P *< 0.05) as compared to that without PHI treatment. The protein expression of *p15 *was examined by Western blotting in parallel. Figure 1C depicts convincingly a dose-related increase of *p15 *expression after treatment with PHI at 20-40 μM, similar to the 5-Aza effect. The results thus indicated that PHI might be a potent methylation inhibitor for the CpG island of *p15 *gene, leading to reactivating *p15 *transcription in the leukemic cells.

### PHI decreased the expression of DNMT1, DNMT3B

**Figure 2:**
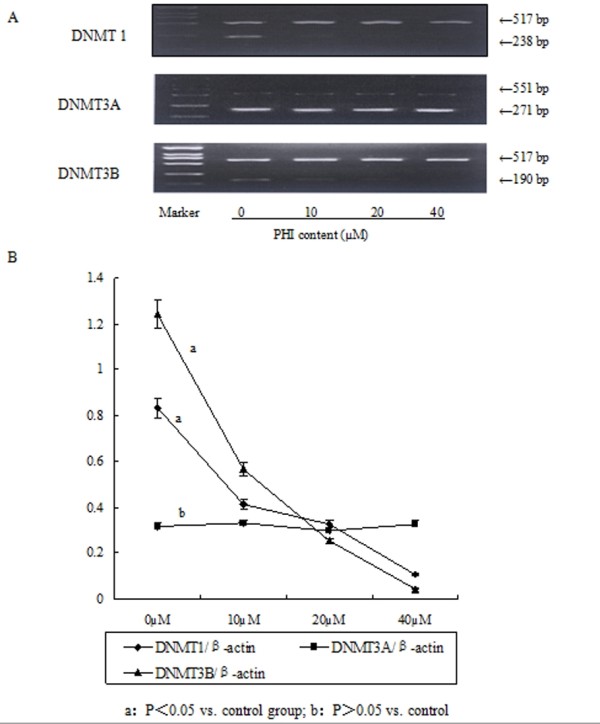
**Down-regulation of the DNA methylating enzymes by PHI**. **A**: The mRNA of DNMT1 and DNMT3B was down regulated by PHI in concentration-dependent manner. **B**: Ratio of the gray scale of DNMTs contrasted to that of β-actin.

The mRNA levels of the enzymes responsible for DNA methylation, i.e., DNMT1, DNMT3A, DNMT3B, without or with the exposure to PHI, were evaluated with RT-PCR (Figure 2A). The gray scale of DNMTs was contrasted to that of β-actin (Figure 2B), and demonstrated that the mRNA of DNMT1 and DNMT3B were significantly decreased after exposure for to PHI for 5 days, in a concentration-dependent manner (*P *< 0.05). The mRNA level of DNMT3A, on the other hand, was not significantly altered under the same condition.

### PHI induced histone acetylation and acetyltransferase up-regulation

**Figure 3:**
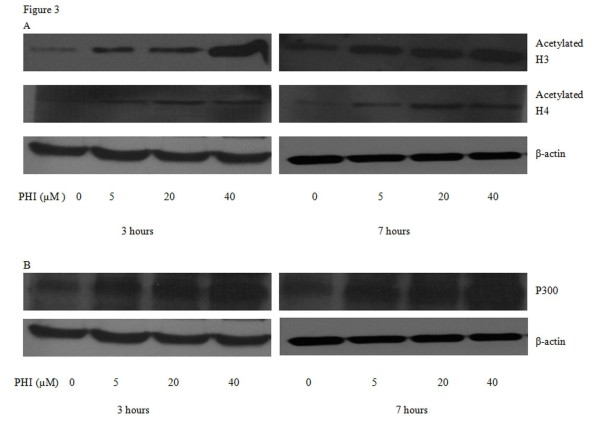
**PHI induced histone acetylation and acetyltransferase up-regulation**. **A**: The acetylation of histone H3 and H4 was significantly increased in a concentration and time-dependent manner by PHI. **B**: PHI up-regulated the expression of acetyltransferase P300/CBP in a concentration and time-dependent manner.

Molt-4 cells were exposed to PHI at various concentrations. Figure 3A showed that after exposure of Molt-4 cells to PHI, the acetylation of histone H3 or H4 was significantly increased in a concentration and time-dependent manner. Acetylated histone H3 was elevated approximately 1.13, 1.21 and 1.35-folds with PHI at 5, 20, or 40 μM for 3 h, as compared to controls without PHI. After 7 hours, acetylation was increased by 2.0, 2.2, 4.0-folds. Similarly, acetylated histone H4 was increased approximately 1.09, 1.45 and 1.72-folds after 3 hours, and 1.3, 1.8, 1.9-folds after 7 hours. The enzyme level of P300/CBP was examined in parallel. Figure 3B showed that the increase of P300/CBP could be clearly observed after exposed to 5 μM or more PHI, similar to the effects on histone acetylation. Approximately 1.1, 1.13 and 1.23-folds increase of P300/CBP, over the control, was observed 3 hours after exposure to PHI at 5, 20 and 40 μM, and approximately 1.38, 1.9 and 2.14-folds after 7 hours.

## Discussion

DNA methylation and histone acetylation are two well known epigenetic chromatin modifications. At least 80 clinical trials are underway, testing more than eleven different HDAC inhibitory agents including both hematological and solid malignancies [23-25]. This study showed for the first time that PHI had dual effects on enhancing histone acetylation as well as demethylating *p15*. Upon exposure to PHI, the unmethylated *p15*, otherwise absent, became detectable, along with the disappearance of the methylated *p15*. This reversal of p*15 *methylation correlates with reexpression of *p15*. The activity of PHI in demethylating *p15 *was shown to be similar to that of 5-Aza and TSA. To our knowledge, this study is the first to demonstrate that the hypermethylated *p15 *gene in leukemic T cells could be reactivated by an isothiocyanate. Our analyses have provided two inter-related mechanisms for hypomethylation of *p15*. They are presented in the following.

One potential mechanism for initiating demethylation of *p15 *by PHI could be that the expressions of the DNA methylating enzymes were down-regulated. This possibility was examined with the three DNA methylating enzymes DNMT1, DNMT3A, and DNMT3B. The study showed that in the presence of PHI, the mRNA of two of the enzymes, DNMT1 and DNMT3B, was significantly down-regulated in a concentration-dependent manner. The mRNA of the enzyme DNMT3A, however, did not show a significant alteration. The expression of DNMT3A is known to be ubiquitous and this has been under investigation as a basis of this observation. The results suggested that reduction of the DNMT1 and DNMT3B expression and their activity could be a responsible mechanism for hypomethylation of *p15*. This interpretation is in line with the reports that DNMTs are commonly over-expressed in leukemias. AML cells with hypermethylated *p15 *tended to express higher levels of DNMT1 and DNMT3B [5,6]. Melki et al [5] demonstrated that the average expression of DNA methyltransferase mRNA from the bone marrow cells of leukemic patients is elevated 4.4-folds, as compared to the expression of normal bone marrow. Mizuno[6] found a mean increase of 5.3-, 4.4-, and 11.7-folds of DNMT1, 3A, and 3B in AML, respectively, comparing with the control bone marrow cells. Although CML cells in the chronic phase did not show significant changes, cells in the acute phase showed 3.2-, 4.5-, and 3.4- fold mean increases in the levels of DNMT1, 3A, and 3B, respectively.

The major mechanism of the current hypomethylating drugs, such 5-Azacytidine and decitabine, is their covalent binding to the DNMTs, which resulting in the irreversible inhibition of the DNMT activity, leading to the hypomethylation of the genomic DNA [26]. Our data showed that PHI could reduce the synthesis of DNMTs. It may be an additional mechanism for PHI to induce DNA hypomethylation. Whether PHI also bind to DNMTs remains to be investigated.

The methylation of *p15 *by PHI could also be related to the PHI effects on histone acetylation. Histone acetylation generally correlates to an open and transcriptionally active chromatin, whereas histone deacetylation is associated with chromatin condensation and transcriptional repression. The *p15 *CpG island region is surrounded with both histone acetylated H3 and methylated H3K9 in AML[27]. PHI could up-regulate the expression of acetyltransferase P300/CBP and induces the accumulation of acetylated histone H3, H4 in Molt-4, resulting in chromatin unfolding and accessibility of regulators in the *p15 *promoter for transcriptional activation. This is consistent with previous data showing that PHI was inhibitor of HDACs [28].

## Conclusion

PHI could induce DNA demethylation and acetylated H3, H4 accumulation in Molt-4 cells. Hypermethylation of gene *p15 *was reversed and *p15 *transcription could be reactivated de novo by PHI.

## List of abbreviations

PHI: Phenylhexyl Isothiocyanate; HDAC: histone deacetylases; MSP: Methylation Specific PCR; 5-Aza: 5-azacytidine; TSA: Trichostatin A; DNMT: DNA methyltransferase; RT-PCR: reverse transcriptase-polymerase chain reaction.

## Competing interests

The authors declare that they have no competing interests.

## Authors' contributions

XM is responsible for study design, writing paper and has been involved in all aspects of this study. SJ, YH, YX and RZ contributed to collecting and analyzing data. Dr Jen-Wei contributed to the study design and manuscript preparation. All authors have read and approved the final manuscript.
